# A novel integrated miRNA–donor age signature enables detection of cardiac allograft vasculopathy

**DOI:** 10.1002/ctm2.70636

**Published:** 2026-03-19

**Authors:** Irene González‐Torrent, Carlota Benedicto, Marta Delgado‐Arija, Lorena Pérez‐Carrillo, Isaac Giménez‐Escamilla, Estefanía Tarazón, Esther Roselló‐Lletí

**Affiliations:** ^1^ Clinical and Translational Research in Cardiology Unit Health Research Institute Hospital La Fe (IIS La Fe) Valencia Spain; ^2^ CB16/11/00261 Group Center for Biomedical Research Network on Cardiovascular Diseases (CIBERCV) Madrid Spain

1

Dear Editor,

Cardiac allograft vasculopathy (CAV) remains a major cause of long‐term mortality after heart transplant (HT).^1^ Current invasive diagnostic methods have limited sensitivity in the early stage of CAV and underestimate the severity of the disease.^2^, ^3^, ^4^ To address that, we conducted a non‐targeted transcriptomic study focused on identifying circulating miRNAs as potential non‐invasive biomarkers for CAV detection at 1‐year surveillance.

In the discovery phase, a total of 70 plasma samples from patients who underwent HT were analysed using RNA‐seq technology (57 without CAV and 13 with CAV). Subsequently, the cohort was expanded to 103 patients for validation using RT‐qPCR. Patients with CAV were classified into low‐grade CAV (CAV_1_, *n* = 6) and high‐grade CAV (CAV_2‐3_, *n* = 9). The clinical characteristics of the patients included at the time of sample collection are summarised in Table 1. Both groups were similar, except for donor age in the validation phase (*p* < 0.05).

**TABLE 1:** Patient characteristics at the time of blood sample extraction.

	Discovery Phase (RNA‐seq)			Validation Phase (RT‐qPCR)		
	No CAV (*N* = 57)	CAV (*N* = 13)	*p*	No CAV (*N* = 88)	CAV (*N* = 15)	*p*
Age (years)	53 (45–61)	58 (52–62)	0.218	54 (46–63)	57 (50–61)	0.653
Male sex, *N* (%)	43 (75%)	11 (85%)	0.718	70 (80%)	12 (80%)	1.000
Indication for cardiac Tx			0.868			0.731
DCM, *N* (%)	20 (35%)	5 (38%)		35 (40%)	5 (33%)	
ICM, *N* (%)	18 (32%)	6 (46%)		28 (32%)	7 (47%)	
Other, *N* (%)	19 (33%)	2 (16%)		25 (28%)	3 (20%)	
Body mass index (kg/m^2^)	25 (21–27)	24 (22–26)	0.975	24 (22–26)	24 (22–26)	0.687
Hypertension, *N* (%)	31 (54%)	5 (38%)	0.365	45 (51%)	6 (40%)	0.578
Diabetes mellitus, *N* (%)	21 (37%)	7 (54%)	0.349	39 (44%)	7 (47%)	1.000
Dyslipemia, *N* (%)	28 (49%)	6 (46%)	1.000	42 (48%)	8 (53%)	0.783
Ventricular assist device before Tx, *N* (%)	28 (49%)	6 (46%)	1.000	40 (45%)	8 (53%)	0.589
CMV infection, *N* (%)	12 (21%)	1 (7.7%)	0.437	18 (20%)	2 (13%)	0.730
Immunosuppressive therapy						
Tacrolimus, *N* (%)	56 (98%)	13 (100%)	1.000	87 (99%)	15 (100%)	1.000
Mycophenolic acid, *N* (%)	55 (96%)	13 (100%)	1.000	86 (98%)	15 (100%)	1.000
Steroids, *N* (%)	55 (96%)	13 (100%)	1.000	85 (97%)	15 (100%)	1.000
Everolimus, *N* (%)	3 (5.3%)	2 (15%)	0.230	4 (4.5%)	2 (13%)	0.210
Neutrophils (thousands/mm^3^)	3.4 (2.3–5.4)	2.8 (2.3–4.9)	0.667	3.6 (2.6–5.3)	3.0 (2.4–5.0)	0.607
Leukocytes (thousands/mm^3^)	6.2 ± 2.6	5.9 ± 1.7	0.664	6.3 (4.7–8.3)	5.7 (4.7–7.8)	0.723
Lymphocytes (thousands/mm^3^)	1.5 (1.2–2.2)	1.6 (1.3–2.0)	0.586	1.6 (1.2–2.1)	1.7 (1.4–2.0)	0.425
Total cholesterol (mg/dl)	146 ± 25	132 ± 33	0.089	149 ± 29	137 ± 31	0.156
HDL (mg/dl)	49 (39–57)	52 (42–58)	0.599	47 (39–53)	52 (43–59)	0.154
LDL (mg/dl)	74 ± 20	61 ± 22	0.069	74 (62‐90)	70 (50‐83)	0.239
Triglycerides (mg/dl)	102 (82–150)	79 (75–133)	0.213	105 (82–147)	80 (76–133)	0.223
Haemoglobin (mg/dl)	12 ± 1.8	13 ± 2.2	0.305	13 ± 2.0	13 ± 2.1	0.647
Hematocrit (%)	40 (34–42)	40 (38–45)	0.231	39 ± 5.9	40 ± 6.6	0.533
NT‐proBNP (pg/ml)	319 (165–682)	231 (126–446)	0.314	327 (172–557)	242 (153–363)	0.201
Troponin T (ng/L)	13 (9.9–44)	13 (10–22)	0.664	13 (10–39)	13 (10–22)	0.602
Creatinine (mg/dl)	1.0 (0.8–1.2)	1.0 (0.8–1.4)	0.506	1.0 (0.8–1.2)	1.0 (0.8–1.4)	0.888
Donor age	46 (31–54)	51 (47–55)	0.158	47 (32–53)	52 (49–58)	0.041

Categorical variables are presented as the number of patients (*N*) and percentage (%), continuous variables as mean ± standard deviation, and non‐continuous variables as median and interquartile range. ACR, acute cellular rejection; CAV, cardiac allograft vasculopathy; CMV, cytomegalovirus; DCM, idiopathic dilated cardiomyopathy; HDL, high‐density lipoprotein; ICM, ischemic cardiomyopathy; LDL, low‐density lipoprotein; NT‐proBNP, N‐terminal fragment of B‐type natriuretic peptide; RNA‐seq, RNA sequencing; RT‐qPCR, reverse transcription‐quantitative polymerase chain reaction; Tx, transplantation.

Large‐scale screening identified nine miRNAs significantly upregulated in patients with CAV, all of which demonstrated good diagnostic performance with area under the curve (AUC) values greater than 0.700 (Figure S1A–C). Cubic spline plots revealed a near‐linear association between these miRNAs and CAV risk, with all microRNAs showing a significant overall effect (*p* < 0.05) except miR‐374b‐5p (Figure S1D). The upregulation of six miRNAs in patients with CAV was validated using RT‐qPCR: miR‐21‐5p (FC = 2.27, *p* < 0.01) (Figure 1A), miR‐223‐3p (FC = 3.34, *p* < 0.001) (Figure 1B), miR‐23a‐3p (FC = 1.43, *p* < 0.05) (Figure 1C), miR‐23b‐3p (FC = 1.53, *p* < 0.05) (Figure 1D), miR‐374b‐5p (FC = 1.49, *p* < 0.05) (Figure 1E), and miR‐98‐5p (FC = 1.87, p < 0.05) (Figure 1F).

**FIGURE 1:**
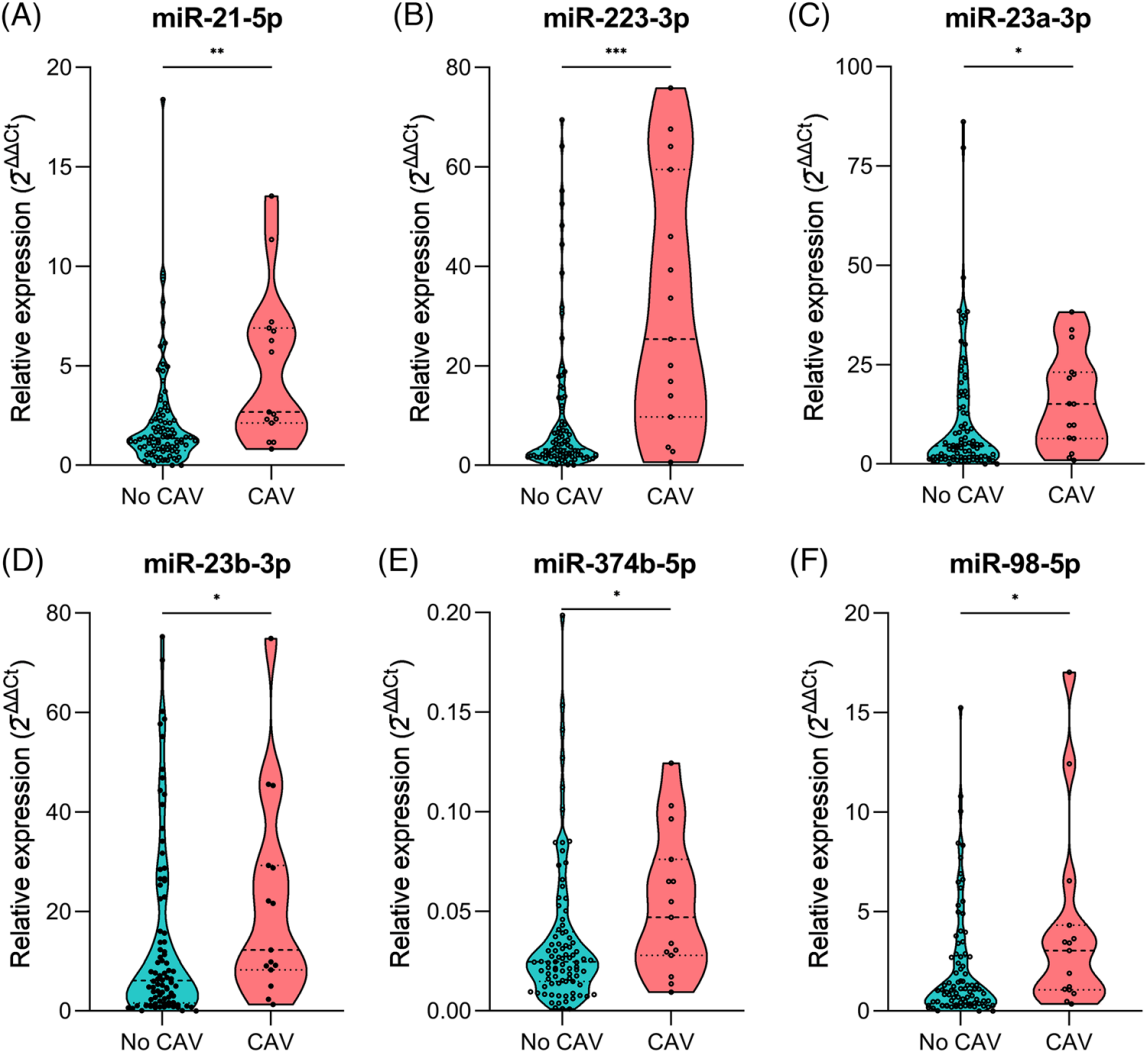
Validation of altered miRNAs in patients with cardiac allograft vasculopathy. Plasma levels of validated miRNAs from patients without cardiac allograft vasculopathy (CAV) and patients with CAV are determined using RT‐qPCR. Violin plots show both the median (dashed line) and the density of the data distribution in each group of the following miRNAs: (A) miR‐21‐5p, (B) miR‐223‐3p, (C) miR‐23a‐3p, (D) miR‐23b‐3p, (E) miR‐374b‐5p, and (F) miR‐98‐5p. * *p* < 0.05, ** *p* < 0.01, *** *p* < 0.001. CAV, cardiac allograft vasculopathy; RT‐qPCR, reverse transcription quantitative polymerase chain reaction.

Receiver operating characteristic (ROC) curves were plotted to analyse the ability of altered miRNAs to detect CAV, and all of them showed a significant AUC (Figure S2A). Based on evidence from our previous study indicating that a combination of miRNAs provides greater diagnostic accuracy than individual miRNAs,^5^ we developed a three‐miRNA signature combining miR‐223‐3p, miR‐23a‐3p, and miR‐23b‐3p. This signature showed higher diagnostic efficacy than individual miRNAs (AUC = 0.850; *p* < 0.001) (Figure S2B).

Moreover, considering that donor age reportedly has a strong association with the disorder^6^ and that it was a clinical variable altered in our cohort with an AUC of 0.665 (*p* < 0.05) (Figure S2C), this variable was incorporated into the model together with the three‐miRNA signature, obtaining an AUC of 0.869 (*p* < 0.001) (Figure S2D). Sensitivity, specificity, positive (PPV) and negative (NPV) predictive values and positive (LR+) and negative (LR‐) likelihood ratios for the diagnosis of CAV for individual miRNAs and their combinations are summarised in Table 2. The combination of the three‐miRNA signature with donor age achieved a sensitivity of 87% and specificity of 81%, and it showed the highest PPV and NPV (45% and 97%, respectively) with superior LR+ (4.7) and lower LR‐ (0.16). After internal validation with bootstrapping, the optimism‐corrected AUC remained stable (corrected AUC = 0.824), supporting model robustness. Overall, this model demonstrated the highest diagnostic accuracy for discriminating between patients with and without CAV.

**TABLE 2:** Receiver operating characteristic (ROC) curve of circulating miRNA candidates for detecting cardiac allograft vasculopathy (CAV).

miRNA	AUC	95% CI	*p*	SS	SP	PPV	NPV	LR+	LR‐
miR‐223‐3p	0.796	0.662–0.931	<0.001	80.0	76.1	36.4	95.7	3.35	0.263
miR‐23a‐3p	0.672	0.534–0.810	0.034	80.0	60.9	26.1	94.6	2.05	0.328
miR‐23b‐3p	0.673	0.544–0.801	0.033	80.0	61.6	26.7	94.6	2.08	0.325
Combined	0.850	0.759–0.942	<0.001	86.7	68.6	32.5	96.7	2.76	0.194
Combined + donor age	0.869	0.759–0.979	<0.001	86.7	81.4	44.8	97.2	4.66	0.163

Sensitivities, specificities and predictive values (%) and likelihood ratios for the diagnosis of cardiac allograft vasculopathy (Cut‐off Point Youden index). AUC, area under the curve; CAV, cardiac allograft vasculopathy; CI, confidence interval; LR+, positive likelihood ratio; LR‐, negative likelihood ratio; NPV, negative predictive value; PPV, positive predictive value; ROC, receiver‐operating characteristic; SS, Sensitivity; SP, specificity.

We observed that our model could detect CAV independently of disease severity. The combination of the three‐miRNA signature with donor age achieved an AUC of 0.783 (*p* < 0.05) for detecting CAV_1_ and an AUC of 0.917 (*p* < 0.001) for detecting CAV_2‐3_ (Figure 2A,B). In addition, the model demonstrated the ability to distinguish patients with high‐grade CAV from those with low‐grade CAV, with an AUC of 0.944 (*p* < 0.01; Figure 2C). Sensitivity, specificity, PPV, NPV, LR+, and LR‐ for distinguishing different severity grades of CAV are detailed in Table S1.

**FIGURE 2:**
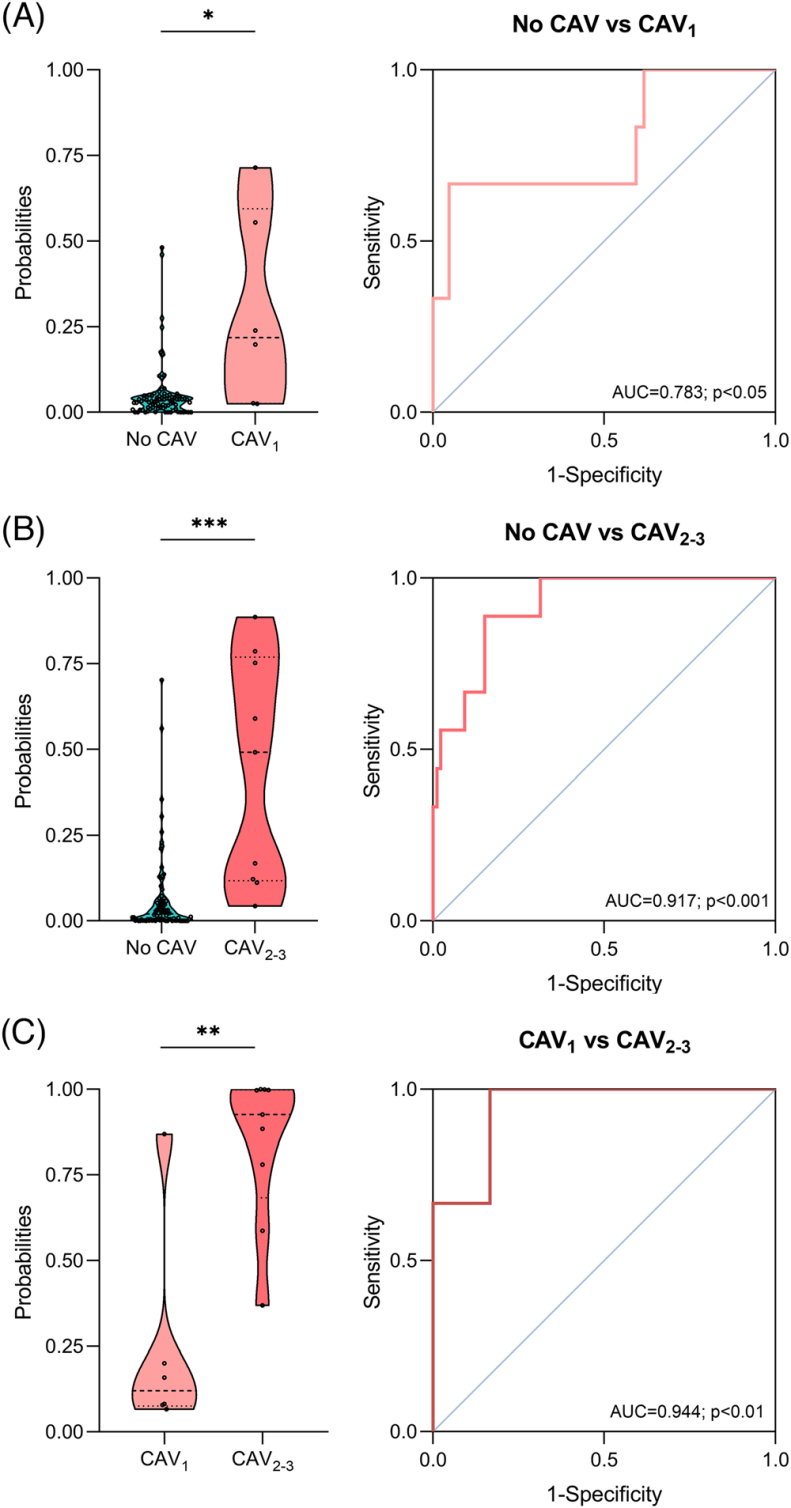
Capacity of the combined model (3‐miRNA signature with donor age) to discriminate between different grades of cardiac allograft vasculopathy (CAV). Predicted probabilities from binary logistic regression models differentiating between (A) No CAV group vs. low‐grade CAV (CAV_1_), (B) No CAV group vs. high‐grade CAV (CAV_2‐3_), and (C) low‐grade vs. high‐grade CAV. The corresponding ROC curve is shown on the right of each violin plot. Violin plots show both the median (dashed line) and the density of data distribution in each group of the predicted probability in the more clinically severe CAV group of the comparison.

Furthermore, to assess whether the combination of the three‐miRNA signature and donor age is related to low‐ and high‐grade CAV, binary logistic regression analysis was performed, adjusting for age and sex. Therefore, our model was independently associated with the presence of CAV_1_ with odds ratio of 41.5 (95% CI 5.7–305.0, *p* < 0.001) and a C statistic of 0.741 (95% CI 0.455–1.000, *p* < 0.05) and for the presence of CAV_2‐3_ with odds ratio of 45.2 (95% CI 5.2–395.1, *p* < 0.001) and a C statistic of 0.852 (95% CI 0.663–1.000, *p* < 0.001).

Our study has several limitations. Although the number of cases identified in our cohort aligns with the expected incidence of CAV at 1‐year post‐heart transplantation (≈8%),^7^ the absolute number of affected patients is small, a limitation inherent to the low frequency of this complication in early follow‐up and the single‐centre nature of the study. Nevertheless, the number of events is comparable to that of previously published research in this field.^7^ Therefore, while the results obtained are consistent and methodologically robust, the model should be interpreted with caution. Overall, our findings provide a solid foundation and constitute a necessary first step to encourage future studies that overcome these limitations, particularly through validation in larger cohorts.

In conclusion, this study identifies a circulating miRNA signature associated with CAV and demonstrates that combining miR‐223‐3p, miR‐23a‐3p, and miR‐23b‐3p levels with donor age provides significant discriminatory ability to detect CAV. The proposed approach may represent a promising non‐invasive strategy to complement invasive coronary angiography, enhancing diagnostic capabilities for detecting CAV and severity stratification.

## AUTHOR CONTRIBUTIONS

Estefanía Tarazón and Esther Roselló‐Lletí designed and supervised the study; Irene González‐Torrent and Carlota Benedicto conducted experiments; Lorena Pérez‐Carrillo, Marta Delgado‐Arija and Isaac Giménez‐Escamilla acquired and analysed data; Estefanía Tarazón, Esther Roselló‐Lletí and Lorena Pérez‐Carrillo contributed to data interpretation and/or discussion; Irene González‐Torrent wrote the manuscript. All authors reviewed the manuscript.

## CONFLICT OF INTEREST STATEMENT

The authors declare no conflict of interest.

## ETHICS STATEMENT

The study was approved by the Ethics Committee (Biomedical Investigation Ethics Committee of University and Polytechnic Hospital La Fe of Valencia, Spain) and was conducted in accordance with the principles outlined in the Declaration of Helsinki^8^, and all subjects gave written informed consent to participate in the study.

## Supporting information



Supporting Information

## Data Availability

The data that support the findings of this study are available from the corresponding author upon reasonable request.

## References

[1] Nikolova AP , Kobashigawa JA . Cardiac allograft vasculopathy: the enduring enemy of cardiac transplantation. Transplantation. 2019;103(7):1338‐1348. doi:10.1097/TP.0000000000002704 31241553 10.1097/TP.0000000000002704

[2] Spartalis M , Spartalis E , Siasos G . Cardiac allograft vasculopathy after heart transplantation: pathophysiology, detection approaches, prevention, and treatment management. Trends Cardiovasc Med. 2022;32(6):333‐338. doi:10.1016/j.tcm.2021.07.002 34303800 10.1016/j.tcm.2021.07.002

[3] O'Hara PE , Gorrai A , Farr M , et al. Revisiting biomarkers of cardiac allograft vasculopathy: addressing the Achilles heel of heart transplantation. Curr Heart Fail Rep. 2024;21(6):580‐590. doi:10.1007/S11897‐024‐00685‐7 39414739 10.1007/s11897-024-00685-7

[4] Van Dijk BCJ , Bos D , Roest S , et al. Coronary computed tomography angiography in heart transplant patients: current insights and future directions. Transplantation. 2025;109(6):945. doi:10.1097/TP.0000000000005266 39841094 10.1097/TP.0000000000005266 PMC12091219

[5] Pérez‐Carrillo L , Sánchez‐Lázaro I , Triviño JC , et al. Combining serum miR‐144‐3p and miR‐652‐3p as potential biomarkers for the early diagnosis and stratification of acute cellular rejection in heart transplantation patients. Transplantation. 2023;107(9):2064. doi:10.1097/TP.0000000000004622 37606906 10.1097/TP.0000000000004622 PMC10442084

[6] Nagji AS , Hranjec T , Swenson BR , et al. Donor age is associated with chronic allograft vasculopathy after adult heart transplantation: implications for donor allocation. Ann Thorac Surg. 2010;90(1):168. doi:10.1016/J.ATHORACSUR.2010.03.043 20609769 10.1016/j.athoracsur.2010.03.043 PMC3033784

[7] Khush KK , Cherikh WS , Chambers DC , et al. The International Thoracic Organ Transplant Registry of the International Society for Heart and Lung Transplantation: thirty‐sixth adult heart transplantation report — 2019; focus theme: donor and recipient size match. J Heart Lung Transplantation. 2019;38(10):1056‐1066. doi:10.1016/j.healun.2019.08.004 10.1016/j.healun.2019.08.004 PMC6816343 31548031

[8] Macrae DJ . The Council for International Organizations and Medical Sciences (CIOMS) guidelines on ethics of clinical trials. Proc Am Thorac Soc. 2007;4(2):176‐178. doi:10.1513/PATS.200701-011GC 17494727

