# Supplementary material for: A novel integrated miRNA–donor age signature enables detection of cardiac allograft vasculopathy
Source: Clin Transl Med. 2026 Mar 19;16(3):e70636. doi: 10.1002/ctm2.70636 (PMC13093342; doi:10.1002/ctm2.70636)
Supplement: Supplementary file 1 — Supporting Information [file CTM2-16-e70636-s001.docx]

**Supplementary Information**

**A novel integrated miRNA–Donor Age signature enables detection of cardiac allograft vasculopathy**

Irene González-Torrent, PhD^1#^; Carlota Benedicto^1#^; Marta Delgado-Arija, PhD^1,2^; Lorena Pérez-Carrillo, PhD^1,2^; Isaac Giménez-Escamilla, PhD^1,2^; Estefanía Tarazón, PhD^1,2*†^ and Esther Roselló-Lletí, PhD^1,2*†^.

^1^Clinical and Translational Research in Cardiology Unit, Health Research Institute Hospital La Fe (IIS La Fe), Avd. Fernando Abril Martorell 106, 46026 Valencia, Spain. ^2^Center for Biomedical Research Network on Cardiovascular Diseases (CIBERCV), Avd. Monforte de Lemos 3-5, 28029 Madrid, Spain.

*Corresponding authors: esther_rosello@iislafe.es (E.R-L); estefania_tarazon@iislafe.es (E.T); Tel: +34-961-24-66-44 (E.R.L & E.T).

#These authors contributed equally to this work.

†These authors contributed equally to this work.

**SUPPLEMENTARY TABLES**

**Table S1 ROC curve of diagnostic model (3-miRNA signature combined with donor age) for differentiate between degrees of CAV.**

|  | **AUC** | **95% CI** | **p** | **SS** | **SP** | **PPV** | **NPV** | **LR+** | **LR-** |
| --- | --- | --- | --- | --- | --- | --- | --- | --- | --- |
| No CAV vs CAV_1_ | 0.783 | 0.559-1.000 | 0.021 | 66.7 | 95.3 | 50.0 | 97.6 | 14.20 | 0.349 |
| No CAV vs CAV_2-3_ | 0.917 | 0.842-0.992 | <0.001 | 88.9 | 84.9 | 38.1 | 98.6 | 5.89 | 0.131 |
| CAV_1_ vs CAV_2-3_ | 0.944 | 0.823-1.000 | 0.005 | 100 | 83.3 | 90.0 | 100 | 6.00 | 0.000 |

Sensitivities, specificities and predictive values (%) and likelihood ratios for the diagnosis of cardiac allograft vasculopathy (Cut-off Point Youden index). AUC, area under the curve; CAV, cardiac allograft vasculopathy; CI, confidence interval; LR+, positive likelihood ratio; LR-, negative likelihood ratio; NPV, negative predictive value; PPV, positive predictive value; ROC, receiver-operating characteristic; SS, Sensitivity; SP, specificity.

**SUPPLEMENTARY FIGURES**


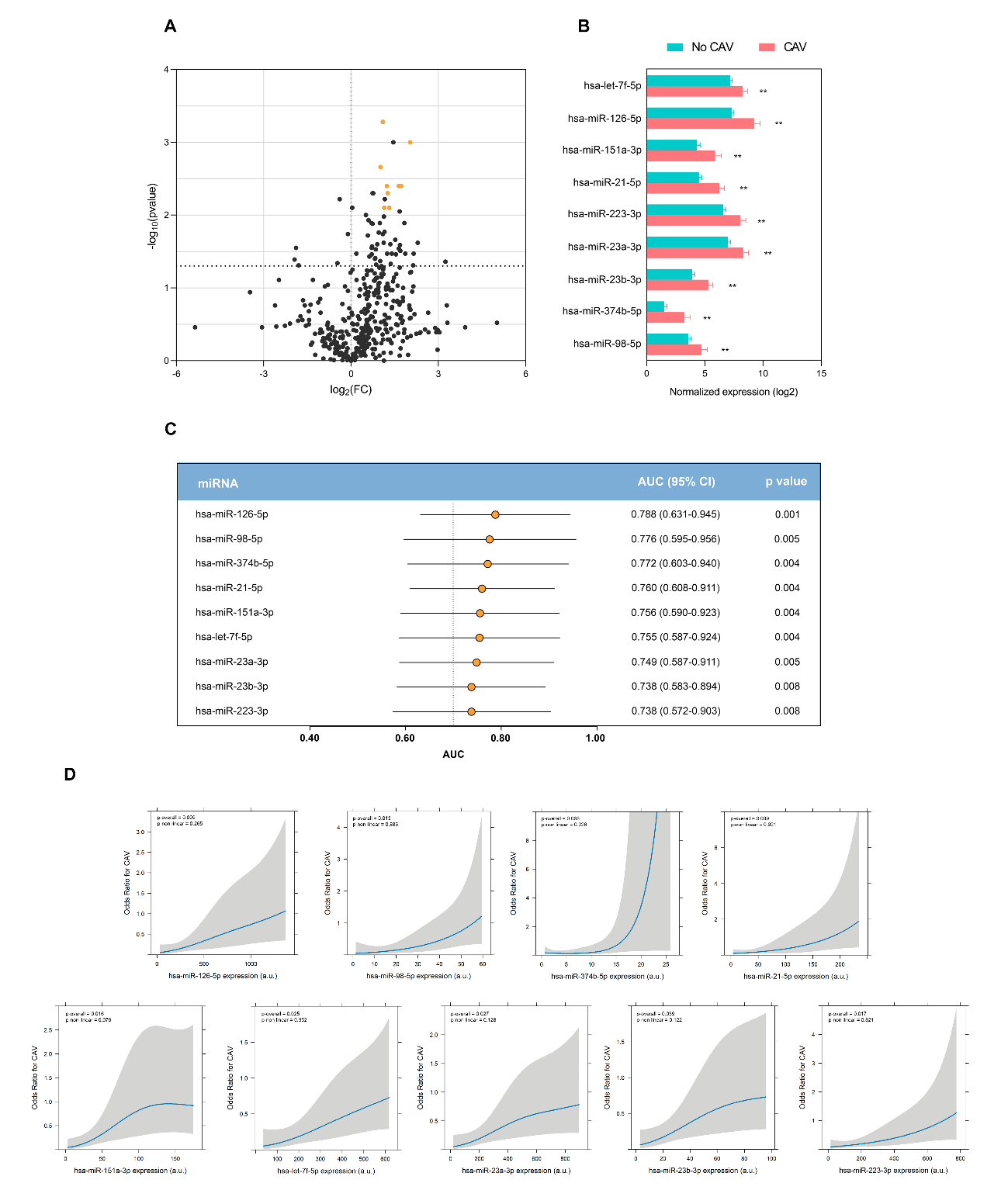


**Figure S1. Screening of differentially expressed miRNAs in the plasma of patients with cardiac allograft vasculopathy.** (A) Volcano plot of differentially expressed miRNAs between patients with and without CAV, determined using RNA-seq. Orange dots indicate altered miRNAs based on the criteria (< 30% missing values and FDR ≥ 0.1). (B) Bar graph comparing normalized expression levels of altered miRNAs between patients with and without CAV. Data are presented as mean ± SEM. ** p < 0.01. (C) Forest plot of the AUC values obtained in the ROC analysis of the altered miRNAs. Data are presented as AUC with 95% CI. (D) Restrictive cubic spline analysis between miRNAs levels and CAV. The odds ratio is represented by the blue line, and the 95% CI by the gray shaded area. AUC, area under the curve; CAV, cardiac allograft vasculopathy; CI, confidence interval; FC, fold change, RNA-seq, RNA-sequencing; ROC, receiver operating characteristic.


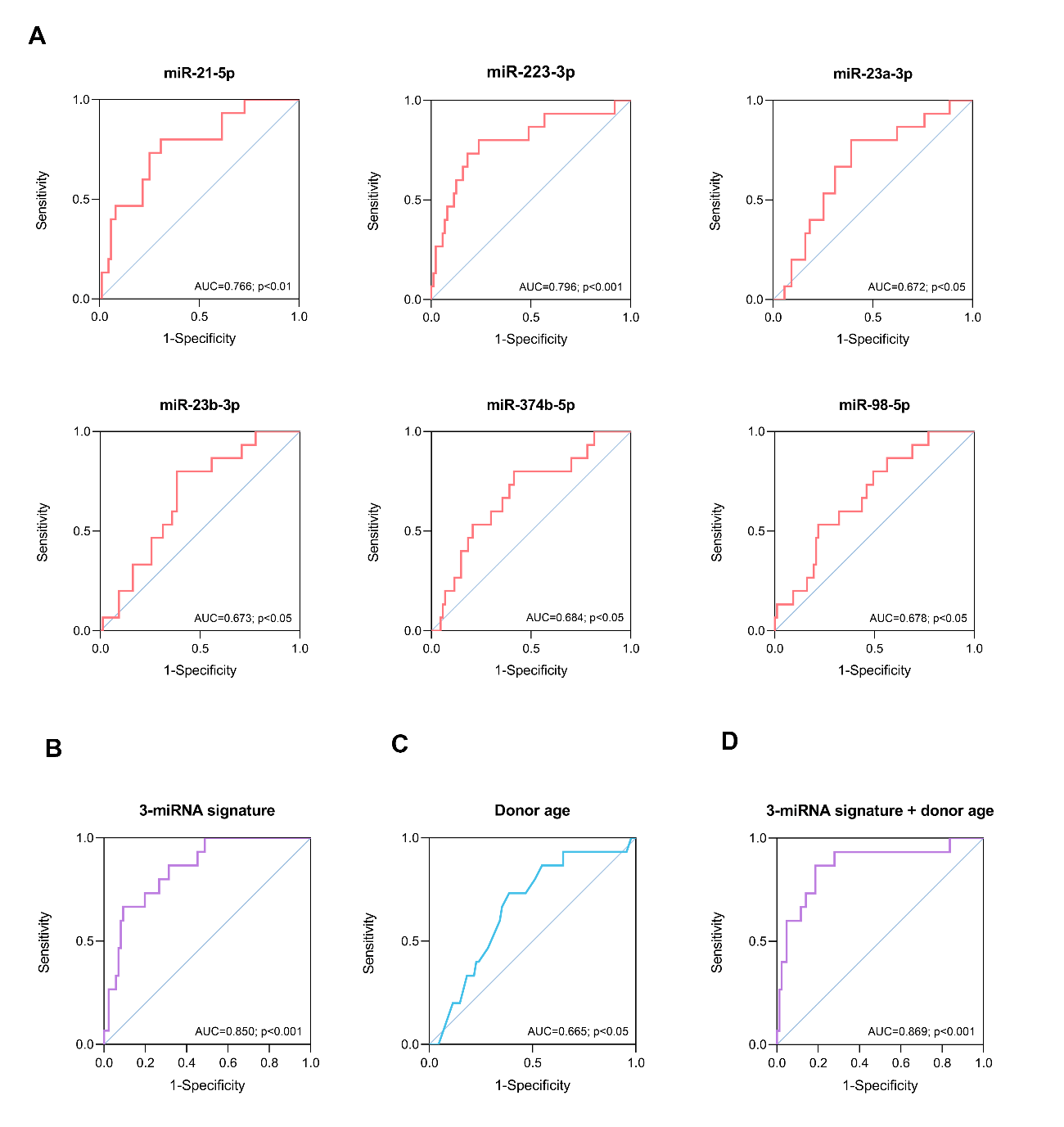


**Figure S2. Receiver operating characteristic analysis of plasma miRNAs for detecting cardiac allograft vasculopathy.** (A) Individual ROC curves of miRNAs. (B) ROC curve of the 3-miRNA signature: combination of miR-223-3p, miR-23a-3p, and miR-23b-3p. (C) ROC curve of donor age. (D) ROC curve of the 3-miRNA signature combined with donor age. AUC, area under the curve. AUC, area under the curve; ROC, receiver operating characteristics.

**SUPPLEMENTARY MATERIAL AND METHODS**

**Sample collection**

In this study, we enrolled patients who underwent HT (aged>18 years) between January 2015 and March 2024 at La Fe University and Polytechnic Hospital. Patients undergoing heart retransplantation or who died in the first year after HT without a CAV diagnosis, were excluded. The diagnosis of CAV was established via coronary angiography performed 1-year post-transplant as part of the clinical protocol and was categorized based on International Society for Heart and Lung Transplantation (ISHLT) classification.^1^

First, this RNA-sequencing (RNA-seq)-based study included 70 patients: 57 without and 13 with CAV. Next, we expanded the cohort to 103 patients, for technical validation using qPCR. For further analysis, we categorized patients with CAV in the validation phase into two groups: low-grade CAV (CAV_1_, n=6) and high-grade CAV (CAV_2-3_, n=9: CAV_2_, n=4; and CAV_3_, n=5). This classification is based on clinical relevance, considering that higher grades have greater risk for major adverse cardiovascular events (MACE), such as death, retransplantation, and coronary revascularization, whereas CAV_1_ has a MACE incidence similar to that of non-CAV.^2,3^

Patients were maintained on a standard immunosuppression regimen, and CAV diagnoses were assessed based on the ISHLT consensus report.^4^ For each sample, we recorded age, sex, body mass index, primary heart disease, interval between transplantation and study enrolment, biochemical markers, and other clinical characteristics at the time of each biopsy (Table 1). The experimenters were blinded to the group allocation and outcome assessment.

Plasma samples and associated clinical data were obtained from the later follow-up visits during the first year after transplantation. A single sample was collected from each patient before coronary angiography. Plasma was separated from the blood samples by centrifugation at 1,500 g for 15 minutes at 4°C, aliquoted, and stored immediately at -80°C.

The study was approved by the Ethics Committee (Biomedical Investigation Ethics Committee of University and Polytechnic Hospital La Fe of Valencia, Spain) and was conducted in accordance with the principles outlined in the Declaration of Helsinki.^5^ Informed consent was obtained from each patient prior to sample collection.

**Discovery phase: plasma RNA-sequencing**

Seventy blood samples were analyzed using plasma RNA-seq. RNA extraction was performed using the miRNeasy® Serum/Plasma Advanced Kit (Qiagen, Hilden, Germany) according to the manufacturer’s instructions. The purity and integrity of the RNA samples were measured using an Agilent Technologies 2100 Bioanalyzer. Complementary DNA (cDNA) libraries were obtained using the SMARTer smRNA-Seq Kit (Illumina, San José, CA, USA) following the manufacturer’s protocol. The libraries were validated by checking their size, purity, and concentration on an Agilent Bioanalyzer. The libraries were pooled in equimolar amounts and sequenced using an Illumina NovaSeq instrument. Image decomposition and quality value calculations were performed using the modules of the Illumina pipeline.

**Validation phase: RT-qPCR**

For validating plasma RNA-seq, 103 blood samples were analyzed. RNA extraction was performed using the miRNeasy® Serum/Plasma Advanced Kit (Qiagen, Hilden, Germany) following the protocol provided by the manufacturer. Total RNA was eluted in 20 µL of RNase-free water. RNA input was normalized using equal volumes of serum (300 µL).

Complementary DNA synthesis was performed with 2 µL of RNA using the TaqMan® Advanced miRNA cDNA Synthesis kit (ThermoFisher Scientific, Whaltam, MA, USA) following the manufacturer’s protocol. Five microliters of the RT reaction product was pre-amplified following the manufacturer’s recommendations. Pre-amplified cDNA was immediately diluted (1/10) and stored at -20 ºC until qPCR.

The reverse transcription reaction product was used for qPCR performed using the TaqMan Gene Expression Assay in a QuantStudio 5 Real-Time PCR System (Applied Biosystems, Waltham, MA, USA) according to the manufacturer’s instructions. The following TaqMan probes were obtained from ThermoFisher Scientific: hsa-let-7f-5p (478578_mir), hsa-miR-126-5p (477888_mir), hsa-miR-151a-3p (477919_mir), hsa-miR-21-5p (477975_mir), hsa-miR-223-3p (477983_mir), hsa-miR-23a-3p (478532_mir), hsa-miR-23b-3p (483150_mir), hsa-miR-374a-5p (478238_mir), and hsa-miR-98-5p (478590_mir). The qPCRs reactions of 20µl were run in 96-well plates by mixing 10µl of Taqman Fast Advanced Master Mix 20X (Applied Biosystems; ThermoFisher Scientific, Carlsbad, CA, USA), 1µL TaqMan Assay 20X, 7µL of RNAse-free water, and 2µL of cDNA (diluted 1/10). qPCR amplifications were performed by incubation at 95 ºC for 10 min, followed by 40 amplification cycles at 95 ºC for 15 s and 60 ºC for 60 s.

For data normalization, hsa-miR-191-5p (477952_mir) was used as an endogenous control. This miRNA was selected as endogenous reference from the discovery phase (RNA-seq) based on its expression level and stability, as described by Pérez-Carrillo et al.^6^ The 2^–∆∆Ct^ method was used to compare relative expression of the miRNAs between samples from the different groups.^7^

**Statistical analysis**

Clinical characteristics are expressed as mean ± standard deviation for continuous variables, as median and interquartile range when presenting a non-normal distribution, and as percentages for discrete variables. The normality of each variable was assessed using the Shaphiro–Wilk test. Continuous variables not following a normal distribution were compared using the Mann–Whitney test, and variables with a normal distribution were compared using Student’s t-test. Fisher’s exact test was used to compare discrete variables. For plasma RNA-seq analysis, we first removed miRNAs with ≥ 30% missing values. Next, we used the false discovery rate (FDR) method to adjust the original p-value using the number of tests. To prevent the identification of false positives in the differential expression data, only differentially expressed miRNAs with a p-value (*P* adj) corrected by FDR ≤ 0.1 were selected as first approximation for the identification of candidate miRNAs.

We constructed a receiver operating characteristic (ROC) curve to evaluate the diagnostic capability of plasma biomarkers for CAV. The area under the ROC curve (AUC) was reported with a 95% confidence interval (CI). The optimal diagnostic point of a miRNA was defined at the cutoff value with the highest Youden index (sensitivity + specificity - 1). Restricted cubic spline regression (RCS) was used to flexibly model and determine whether a nonlinear relationship existed between miRNA levels and CAV. With the validation results, binary logistic regression analysis was performed, combining different miRNAs and clinical variables and adjusting the model for age and sex. To ensure robust internal validation, bootstrap resampling (1000 iterations) was performed, estimating optimism-corrected AUC. Statistical significance was set at p<0.05. All statistical analyses were performed using R (version R-4.3.1), SPSS (version 20.0), and GraphPad Prism (version 8.0).

**EXTENDED DISCUSSION**

CAV remains highly prevalent and a leading cause of mortality after HT.^4^ Coronary angiography is the current gold standard practice for CAV surveillance in HT recipients, despite the risk of severe complications it carries, including contrast-related kidney injury and procedure-related vascular lesions.^25^ Moreover, ICA has some technical limitations; it only provides information regarding the vessel lumen and may miss early stages of CAV, which occur as changes of the coronary wall.^26^ Other invasive techniques, IVUS and OCT, can supplement ICA to improve its sensitivity. However, they present certain limitations for routine surveillance of CAV, such as high cost, availability, anticoagulation requirement, associated risks, and evaluation limited to the major epicardial vessels.^27^ Non-invasive imaging techniques are also used for CAV surveillance, but demonstrate limited sensitivity for detecting less severe ISHLT grade CAV_1_ and early coronary intimal thickening.^27^ Therefore, there is a need to develop a non-invasive method that complements ICA in achieving optimal sensitivity for CAV diagnosis, particularly in the early phase, and in discriminating between the different grades of severity.

In this context, blood-based biomarkers would be an interesting non-invasive source of information for CAV progression, as has been demonstrated in ACR surveillance.^27^ Methods included in clinical practice to monitor allograft rejection have been studied to determine their usefulness in detecting CAV. Specifically, BNP level was correlated with the development of CAV in the late post-transplant period. However, because of its highly variable levels as a result of other conditions, it is not specific enough to provide clinically useful information.^1^ Meanwhile, Yamani et al. described that an increased Allomap score was related to CAV;^8^ however, a recent multicenter study with a larger cohort, reported no association between higher score and CAV.^9^ The association between dd-cfDNA and CAV also remains inconclusive, as studies have reported contradictory findings.^10^ Furthermore, other proteins have been investigated in plasma, but none of them have been translated into clinical practice. Przybylek et al. developed a cytokine score for detecting advanced CAV, combining inflammatory markers that were not altered in patients with CAV.^11^ The score’s complexity makes it challenging for clinical use.^12^ Daly et al., reported that a combination of angiogenesis-related proteins (vascular endothelial growth factor (VEGF)-C, VEGF-A, and platelet factor 4) showed high sensitivity and specificity for CAV diagnosis; but the validation has not been performed yet.^13^ Additionally, Wei et al. identified an urinary proteomic signature with potential utility in CAV diagnosis, but not in discriminating different grades of CAV.^14^

MicroRNAs exhibit biological and technical qualities that make them excellent clinical biomarkers. They can be easily detected in blood with standard clinical laboratory techniques such as qPCR and remain stable across temperature changes.^15,16^ Several studies reported the usefulness of miRNAs for diagnosing ACR after HT.^6,17,18^ However in the context of CAV, the role of these molecules has been less studied. In 2015, Singh et al. found that miR-92a-3p and miR-126-5p combined with age and creatine could discriminate between patients with and without CAV.^19^ However, the plasma miR-126-5p levels were not significantly increased in patients with CAV compared to patients without CAV, which is in accordance with our validation phase results. In 2017, Neumann et al. proposed miR-628-5p as a new potential biomarker of CAV.^20^ However, neither of these biomarkers have been prospectively validated to determine if miRNA can detect incipient CAV.^21^ Moreover, both studies selected miRNAs for further analysis based on literature, which biased the results. In contrast, in 2021, Usuelli et al. identified miRNAs altered in the heart samples of patients with CAV using RNA-seq, a non-targeted method.^22^ They demonstrated that miR-21-5p was significantly upregulated in CAV, but its diagnostic capacity was not explored. Consistent with their findings, we observed elevated levels of miR-21-5p in patients with CAV.

In this study, we identify 9 miRNAs, including miR-21-5p, that were significantly overexpressed in patients with CAV compared to patients without CAV, using RNA-seq. For the validation phase, we amplified the cohort of patients used in the discovery phase and corroborated the overexpression of 67% of the miRNAs: miR-21-5p, miR-223-3p, miR-23a-3p, miR-23b-3p, miR-374b-5p, and miR-98-5p. Among them, miR-223-3p achieved the highest AUC as well as sensitivity and specificity of any single miRNA. Considering evidence from our previous study that suggested that a combination of miRNAs offers greater diagnostic accuracy than single miRNAs,^6^ we developed a 3-miRNA signature combining miR-223-3p, miR-23a-3p, and miR-23b-3p, which showed higher diagnostic efficacy than individual miRNAs.

Numerous studies have reported that advanced donor age is an independent risk factor of CAV development.^23,24^ As the donors of patients with CAV were older than donors of patients without CAV in our cohort, we incorporated donor age into our model together with the 3-miRNA signature. This combination revealed a better capacity for detecting CAV with high sensitivity (87%), specificity (81%), and negative predictive value (97%). The relatively modest positive predictive value observed in our model is largely driven by the low prevalence of CAV in the study population. Conversely, the high negative predictive value (97%) suggests that the model may be particularly suited as a rule-out strategy rather than a rule-in test. In this context, the proposed approach could serve as a non-invasive screening adjunct to coronary angiography. Moreover, despite the small sample size, our results suggest potential discriminatory value of this combination for detection of patients with CAV of any grade and discriminate between low-grade and high-grade CAV. This could have important implications for their use in CAV surveillance, as grading of CAV is associated with long-term outcomes following HT. Those with both moderate and severe CAV have a greater risk for cardiovascular complications, such as death, acute coronary syndrome, coronary revascularization, and cardiac retransplantation.^2,3,25^

**REFERENCES**

1. Mehra MR, Crespo-Leiro MG, Dipchand A, et al. International Society for Heart and Lung Transplantation working formulation of a standardized nomenclature for cardiac allograft vasculopathy-2010. *Journal of Heart and Lung Transplantation*. 2010;29(7):717-727. doi:10.1016/j.healun.2010.05.017

2. Van Keer JM, Van Aelst LNL, Rega F, et al. Long-term outcome of cardiac allograft vasculopathy: Importance of the International Society for Heart and Lung Transplantation angiographic grading scale. *Journal of Heart and Lung Transplantation*. 2019;38(11):1189-1196. doi:10.1016/j.healun.2019.08.005

3. Prada-Delgado O, Estvez-Loureiro R, Paniagua-Martn MJ, Lpez-Sainz A, Crespo-Leiro MG. Prevalence and prognostic value of cardiac allograft vasculopathy 1 year after heart transplantation according to the ISHLT recommended nomenclature. *Journal of Heart and Lung Transplantation*. 2012;31(3):332-333. doi:10.1016/j.healun.2011.12.006

4. Stewart S, Winters GL, Fishbein MC, et al. Revision of the 1990 working formulation for the standardization of nomenclature in the diagnosis of heart rejection. *Journal of Heart and Lung Transplantation*. 2005;24(11):1710-1720. doi:10.1016/j.healun.2005.03.019

5. Macrae DJ. The Council for International Organizations and Medical Sciences (CIOMS) guidelines on ethics of clinical trials. *Proc Am Thorac Soc*. 2007;4(2):176-178. doi:10.1513/PATS.200701-011GC

6. Pérez-Carrillo L, Sánchez-Lázaro I, Triviño JC, et al. Combining Serum miR-144-3p and miR-652-3p as Potential Biomarkers for the Early Diagnosis and Stratification of Acute Cellular Rejection in Heart Transplantation Patients. *Transplantation*. 2023;107(9):2064. doi:10.1097/TP.0000000000004622

7. Livak KJ, Schmittgen TD. Analysis of relative gene expression data using real-time quantitative PCR and the 2-ΔΔCT method. *Methods*. 2001;25(4):402-408. doi:10.1006/METH.2001.1262

8. Yamani MH, Taylor DO, Rodriguez ER, et al. Transplant Vasculopathy Is Associated With Increased AlloMap Gene Expression Score. *Journal of Heart and Lung Transplantation*. 2007;26(4):403-406. doi:10.1016/j.healun.2006.12.011

9. Moayedi Y, Foroutan F, Miller RJH, et al. Risk evaluation using gene expression screening to monitor for acute cellular rejection in heart transplant recipients. *Journal of Heart and Lung Transplantation*. 2019;38(1):51-58. doi:10.1016/j.healun.2018.09.004

10. Chih S, Chong AY, Mielniczuk LM, Bhatt DL, Beanlands RSB. Allograft Vasculopathy the Achilles’ Heel of Heart Transplantation. *J Am Coll Cardiol*. 2016;68(1):80-91. doi:10.1016/j.jacc.2016.04.033

11. Przybylek B, Boethig D, Neumann A, et al. Novel Cytokine Score and Cardiac Allograft Vasculopathy. *American Journal of Cardiology*. 2019;123(7):1114-1119. doi:10.1016/J.AMJCARD.2018.12.034

12. O’Hara PE, Gorrai A, Farr M, et al. Revisiting Biomarkers of Cardiac Allograft Vasculopathy: Addressing the Achilles Heel of Heart Transplantation. *Curr Heart Fail Rep*. 2024;21(6):580-590. doi:10.1007/S11897-024-00685-7

13. Daly KP, Seifert ME, Chandraker A, et al. VEGF-C, VEGF-A and related angiogenesis factors as biomarkers of allograft vasculopathy in cardiac transplant recipients. *J Heart Lung Transplant*. 2013;32(1):120. doi:10.1016/J.HEALUN.2012.09.030

14. Wei D, Trenson S, Van Keer JM, et al. The novel proteomic signature for cardiac allograft vasculopathy. *ESC Heart Fail*. 2022;9(2):1216. doi:10.1002/EHF2.13796

15. Shah P, Bristow MR, Port JD. MicroRNAs in Heart Failure, Cardiac Transplantation, and Myocardial Recovery: Biomarkers with Therapeutic Potential. *Curr Heart Fail Rep*. 2017;14(6):454-464. doi:10.1007/S11897-017-0362-8

16. Koch PF, Ludwig K, Krenzien F, et al. miRNA as potential biomarkers after liver transplantation: A systematic review. *Transplant Rev*. 2024;38(2):100831. doi:10.1016/J.TRRE.2024.100831

17. Shah P, Agbor-Enoh S, Bagchi P, et al. Circulating MicroRNAs in Cellular and Antibody-Mediated Heart Transplant Rejection. *J Heart Lung Transplant*. 2022;41(10):1401. doi:10.1016/J.HEALUN.2022.06.019

18. Constanso-Conde I, Hermida-Prieto M, Barge-Caballero E, et al. Circulating miR-181a-5p as a new biomarker for acute cellular rejection in heart transplantation. *Journal of Heart and Lung Transplantation*. 2020;39(10):1100-1108. doi:10.1016/j.healun.2020.05.018

19. Singh N, Heggermont W, Fieuws S, Vanhaecke J, Van Cleemput J, De Geest B. Endothelium-enriched microRNAs as diagnostic biomarkers for cardiac allograft vasculopathy. *Journal of Heart and Lung Transplantation*. 2015;34(11):1376-1384. doi:10.1016/j.healun.2015.06.008

20. Neumann A, Napp LC, Kleeberger JA, et al. MicroRNA 628-5p as a novel biomarker for cardiac allograft vasculopathy. *Transplantation*. 2017;101(1):e26-e33. doi:10.1097/TP.0000000000001477

21. Pober JS, Chih S, Kobashigawa J, Madsen JC, Tellides G. Cardiac allograft vasculopathy: Current review and future research directions. *Cardiovasc Res*. 2021;117(13):2624-2638. doi:10.1093/CVR/CVAB259

22. Usuelli V, Ben Nasr M, D’Addio F, et al. miR-21 antagonism reprograms macrophage metabolism and abrogates chronic allograft vasculopathy. *American Journal of Transplantation*. 2021;21(10):3280-3295. doi:10.1111/ajt.16581

23. Nagji AS, Hranjec T, Swenson BR, et al. Donor Age Is Associated With Chronic Allograft Vasculopathy After Adult Heart Transplantation: Implications for Donor Allocation. *Ann Thorac Surg*. 2010;90(1):168. doi:10.1016/J.ATHORACSUR.2010.03.043

24. Raichlin E, Edwards BS, Kremers WK, et al. Acute Cellular Rejection and the Subsequent Development of Allograft Vasculopathy After Cardiac Transplantation. *Journal of Heart and Lung Transplantation*. 2009;28(4):320-327. doi:10.1016/J.HEALUN.2009.01.006

25. Kindel SJ, Law YM, Chin C, et al. Improved detection of cardiac allograft vasculopathy: A multi-institutional analysis of functional parameters in pediatric heart transplant recipients. *J Am Coll Cardiol*. 2015;66(5):547-557. doi:10.1016/j.jacc.2015.05.063
